# Flowering seasonality drives taxonomic, functional, and phylogenetic diversity of hummingbirds along an altitudinal gradient in northwestern Mexico

**DOI:** 10.1371/journal.pone.0324881

**Published:** 2025-06-11

**Authors:** Gabriel López-Segoviano, Laura E. Nuñez-Rosas, Maribel Arenas-Navarro, Guillermo Huerta-Ramos, María del Coro Arizmendi

**Affiliations:** 1 Facultad de Estudios Superiores Iztacala, Universidad Nacional Autónoma de México, Tlalnepantla, Estado México, México; 2 Escuela Nacional de Estudios Superiores (ENES) Unidad Morelia, Universidad Nacional Autónoma de México, Morelia, Michoacán, México; 3 Laboratorio de Ecología, UBIPRO, Facultad de Estudios Superiores Iztacala, Universidad Nacional Autónoma de México, Tlalnepantla, Estado México, México; Instituto Federal de Educacao Ciencia e Tecnologia Goiano - Campus Urutai, BRAZIL

## Abstract

Floral resources in the mountains of northwestern Mexico are strongly seasonal. This influences latitudinal, local, and altitudinal migration patterns in hummingbird species, resulting in temporal changes in hummingbird community structure over the flowering period. In this study, we evaluated how the seasonality of floral resources and latitudinal migration of hummingbirds influenced taxonomic, phylogenetic, and functional diversity along an altitudinal gradient. We examined the functional and morphological space occupied by resident and migrant hummingbird species, as well as the species’ functional niches and the phylogenetic structure of species assemblages in three sites of northwestern Mexico. We recorded hummingbird and flower abundance using two transects with 15 point counts on each altitudinal gradient in the Sierra Madre Occidental. The transects spanned from tropical deciduous forest to pine-oak forest and the ecotone between them. We recorded 20 hummingbird species and 70 flowering plant species belonging to 30 families. We found that taxonomic, phylogenetic, and functional diversity were linked to the flower abundance. Seasonal latitudinal hummingbird migration was less important for functional diversity (since migratory species performed redundant functional roles) than taxonomic and phylogenetic diversity. Seasonal flowering along the altitudinal gradient drives various types of hummingbird movements (local, altitudinal, and latitudinal), which increases the number of hummingbird species fulfilling each functional role. Apparent phylogenetic clustering in the ecotone may be due to a convergence between temperate and tropical forest flora and the midpoint of hummingbirds’ altitudinal migrations. Our study highlights the temporal dynamism and strong influence of floral seasonality on the structure of hummingbird communities in the mountains of northwestern Mexico.

## Introduction

The determinants of the presence or absence of species and their coexistence within a community have long been a central question for ecologists. MacArthur and Levins [1] proposed that the coexistence of species in a community depends heavily on their similarity and the abundance of resources. Meanwhile, the abundance of each species within the community is determined by processes that reassemble the random partitioning of resources along a continuum of resource types [1,2]. Thus, the availability of resources and their stability can determine the number of species and their level of specialization/generalization in the community [3]. For instance, regions that are more stable in terms of resource availability, such as the tropics, may support more species and potentially more specialization on particular resources [3].

Hummingbirds are an ideal model system for examining the relationship between resource availability and species abundance due to their high specialization and dependence on a narrow range of resources—namely, floral nectar [4]. This dependence exerts significant fitness pressure on individuals to locate reliable sources of nectar [5]. Supply/demand dynamics between hummingbirds and flowers encourage the evolution of optimal resource use, as hummingbird populations are often at or near their carrying capacity [6]. Hence, the temporal and spatial distribution of nectar has a strong impacts hummingbird assemblages, shaping foraging niches according to each species’ foraging strategy [5–12]. Therefore, hummingbird community structure is driven by the local supply of floral resources [6,13], which is the primary force for their evolution [7].

At the regional level, environmental filtering plays a crucial role in shaping the composition, traits, and structure of hummingbird communities [14,15]. Factors such as temperature, precipitation, and vegetation structure are closely associated with hummingbird species distribution [7,9,14,15]. For example, in the Andes mountains, altitudinal gradients impose a strong ecological filter on the distribution of hummingbirds, since ascending or descending in altitude implies changes in climate and vegetation composition [15].

Hummingbird diversity is indirectly influenced by climate seasonality [7]. For example, the availability of floral resources in the mountains of Mexico is heavily dependent on seasonal precipitation and temperature [16]. Many flowers that hummingbirds feed on bloom after the rainy season in the temperate forest of Northwestern Mexico, which leads to a large increase in hummingbird species and abundance during that season [5,17,18]. Rappole and Schuchmann [5] propose that dependence on a seasonal resource means that each hummingbird species must have unique ways of obtaining the necessary amount of nectar. Some hummingbirds respond by changing their feeding behavior (between territorial and traplining strategies [19]) or migrating (short or large distances [8,20]).

Migratory movements are crucial for the survival and reproduction of hummingbirds, allowing them to find suitable sites to exploit nectar resources [5]. Some species of hummingbirds that breed in the US and Canada migrate to Mexico during the fall or winter [8,21], arriving to areas in northwestern Mexico, such as Concordia in Sinaloa, during the period of abundant flowering [22]. For example, *Selasphorus rufus* breeds in the Rocky Mountains in the northern USA, Canada, and southern Alaska and migrates to winter in El Palmito, Concordia from November through February [8]. The migration of *S. rufus* through northwestern Mexico correlates with the flowering phenology of the most abundant flowering plant visited by resident and migratory hummingbirds in the pine-oak forest, *Salvia iodantha* [8]. Also, *S. rufus* pollinates some flowering plant species and can be considered a generalist core species during the fall/winter at El Palmito, Concordia [23]. Hence, migrant hummingbirds may affect ecosystem functioning in the communities they connect through their journeys. However, understanding their important functions requires integrative studies that link biogeography to community ecology and other disciplines [24].

During the fall and winter, the mountains of Concordia in northwestern Mexico are home to six species of latitudinal migratory hummingbirds; three of these species breed in the USA and Canada, while the other three breed in the USA and the desert region of northern Mexico [21,25]. These migratory hummingbirds integrate into different communities along their migratory routes, thus temporarily increasing the richness and abundance of hummingbirds at the sites where they arrive. For example, *S. rufus* can reach similar abundance to the most abundant resident species in the region—*Basilinna leucotis* and *Saucerottia beryllina—*with which they compete for floral resources [8,26]. Because hummingbird communities experience temporal variation in richness and abundance, it is important to study the impacts of this variation on the region’s taxonomic, functional, and phylogenetic diversity. In this study, we therefore evaluated whether the seasonality of floral resources and latitudinal migratory movements of hummingbirds influenced the taxonomic, phylogenetic, and functional diversity of three hummingbird communities along an altitudinal gradient and determined the functional and morphological space occupied by migratory hummingbirds. Then, we evaluated the functional uniqueness and morphofunctional overlap of each hummingbird species and the phylogenetic structure of species assemblages along an altitudinal gradient. We expected that the arrival of latitudinal migratory hummingbird species would increase the number of species, increasing the taxonomic and phylogenetic diversity of the region. However, since latitudinal migratory species have generalist traits, we expected their morphological and functional niches to overlap with local generalist species, such that their arrival would not necessarily increase the region’s functional diversity.

## Methods

### Study area

The study was conducted in Concordia, Sinaloa. This locality is in the Sierra Madre Occidental (SMO), which is the longest continuous mountain range in Mexico and runs along the Pacific slope. The SMO altitudinal gradient leads to a shift in vegetation type from tropical dry forests in the lowlands to temperate forests—such as oak, conifer, and cloud forests—at higher altitudes [27]. We selected three sites that differ in elevation and vegetation type. The first site was in the lower part of the SMO, between 148 and 289 m a.s.l., and the vegetation there consisted of tropical semideciduous forest, tropical deciduous forest, riparian vegetation, and secondary vegetation (“Tropical”). The second site was located at an intermediate elevation, between 1131 and 1423 m a.s.l. and represented a transition zone (“Ecotone”) between pine-oak, oak, and tropical semideciduous forest; some riparian and secondary vegetation was also present. The third site was at the upper end of the elevation range, between 1800 and 2218 m a.s.l., and mainly consisted of pine-oak (mixture of pine and oak trees, where pines dominate) forest and pine forest, oak-pine (mixture of pine and oak trees, where oaks dominate), cloud forest, and riparian and secondary vegetation (“Pine-oak”).

### Ethics statement

An ethics statement is not required for the present study. No specific permits were required for the described methodology. Our field studies did not involve endangered or protected species, and hummingbird morphological measurements were obtained from museum specimens and previously published manuscripts. No live animals were manipulated.

### Hummingbird and flower abundance

To determine the hummingbird diversity at each site, we established two 3 km-long transects containing 15 point counts separated by 200 m. At each point count, two observers identified all hummingbird species and counted the number of individuals (abundance) during a 10 min period in a 25-m fixed radius around each point count [28]. We did not include hummingbirds that flew over the point count. All point counts were sampled for six days, starting the transect 15 minutes after sunrise and concluding five hours later.

To estimate flower availability, within each of the 25m-radius bird count points, we determined by eye the orientation of the diameter with the highest apparent flower abundance and identified and counted all flowers in a 2m-wide strip centered on that diameter (essentially a 25 m × 2 m transect within each count point). All point counts were sampled once daily over six consecutive days each month. We performed eleven sampling campaigns in one year (one per month) to evaluate plants and hummingbirds from November 2015 to September 2016.

### Functional traits

We used measurements of three hummingbird morphological traits that influence plant-hummingbird interactions—body weight, bill length, and bill curvature—to evaluate species-level functional traits [29,30]. We used data previously published by López-Segoviano et al. [26] measured at the Pine-oak site (El Palmito) supplemented with data measured from specimens at the Museum of Zoology ‘Alfonso L. Herrera’ (MZFC, UNAM) and Colección Nacional de Aves (Instituto de Biología UNAM). Bill length (mm) was measured using a calliper, and body mass was recorded using a digital scale to the nearest 0.10 g. We calculated bill curvatures (°) from photographs of each individual using ImageJ software (http://rsbweb.nih.gov/ij/) [30]. We pooled morphological data for males and females of the species that were recorded because there was insufficient sample size to divide by sex; however, it has been previously shown that sex-related intraspecific variation in hummingbirds is low relative to interspecific variation, even sexually dimorphic species [11,28].

### Migratory status

We use Howell and Webb [25], Arizmendi and Berlanga [21], and Aves.mx [31] to classify hummingbird species by their migratory status. We classified as latitudinal migratory species (S1 Table) those that move between two distinct home ranges in different seasons that are found at different latitudes [5]. The species that moved along the altitudinal gradient during the study and were only recorded briefly at some study sites were classified as altitudinal migratory species (S1 Table). We classified as residents the species that was recorded in over 80% of the samplings at a given site (S1 Table). Local migratory (vagrant) species are species that were neither latitudinal or altitudinal migrants but were only recorded during the blooming period (e.g., *Colibri thalassinus* and *Selasphorus heloisa*) and could therefore be moving between habitats in the same altitudinal belt (S1 Table).

### Diversity measures

We quantified taxonomic, functional, and phylogenetic alpha diversity at each transect for each sampling (eleven months) based on each species’ records. We performed all diversity analyses using the total of the hummingbird species from each sampling, recorded on the two transects containing 15 point counts at each site. To calculate taxonomic diversity, we used species richness (q = 0), which remains the primary measure of biodiversity [32]. We used the mean measures of hummingbird traits (log-transformed body weight, log-transformed bill length, and log-transformed bill curvature) to analyze functional diversity using Rao’s index (*Q*). Rao’s quadratic entropy index of diversity, *Q*, was calculated as the sum of trait dissimilarities between pairs of species multiplied by species abundance (see de Bello et al. [33], Ricotta et al. [34]). For the phylogenetic analysis, we pruned the global hummingbird phylogeny of McGuire et al. [35] to match our dataset. We used the *Picante* [36] package in R to calculate Faith’s phylogenetic diversity (PD) index, which quantifies the total branch length of a phylogenetic tree represented by a community. The taxonomic and phylogenetic alpha diversity index was calculated using the adiv package [37].

### Functional uniqueness

There have been numerous studies assessing the associations between species’ ecological roles and their functional traits and evaluating the likelihood that species with similar traits support similar functions [38,39]. To better explore variation in functional diversity, we quantified functional uniqueness, which refers to how important a species is in supporting a specific function. Species-level functional uniqueness summarizes the functional contribution of a single species to the overall redundancy of the community [34]. The functional dissimilarities between species range from 0 (if all species are functionally identical to a given species) to 1 (if all species are maximally dissimilar to that species) [34]. Low levels of functional uniqueness show redundant morphological traits. We calculated hummingbirds’ species-level functional uniqueness (*K*i) for each site, which gives the mean distance of a given species from all other species in the assemblage [34]. To calculate hummingbird functional uniqueness, we calculated the mean for each species for each site, and the trait matrices were log-transformed before the calculations. To calculate the uniqueness coefficient, we used the script in R software provided by Ricotta et al. [43].

### Morphofunctional space

The functional space was calculated under the *n*-functional hypervolume approach based on Hutchinson’s multidimensional niche concept [40,41]. This approach allowed us to quantify functional space and volume for each hummingbird species [42,43]. To avoid multicollinearity, we performed a principal component analysis (PCA) on the bill length, bill curvature, and body weight of 637 hummingbird individuals [44,45]. We used the first three PC axes to calculate the hypervolume and the Gaussian kernel density estimation with the “Silverman” method to estimate the bandwidth [42]. The morphospace was constructed per site (Pine-oak, Ecotone, and Tropical); we obtained a volume for each species and calculated the overlap between species pairs within each site. This procedure involved comparing the similarity between different hypervolumes using the Sørensen index of functional similarity, which estimates the intersection between two hypervolumes (given two hypervolumes A and B, S (A, B) = 2 * | A \ B |/ (| A | + | B |) ranging from 0 (the two input hypervolumes are completely distinct) to 1 (the two hypervolumes are identical) [42,45]. We used the package “hypervolume” ver. 3.1.0 [40,42].

### Phylogenetic community structure

For the three hummingbird communities, we calculated the Net Relatedness Index (NRI) and Nearest Taxon Index (NTI). NRI is based on the mean phylogenetic distance (MPD), which is calculated from the pairwise cophenetic branch length distances. NRI is calculated by multiplying the median of standardized effect size phylogenetic distances by −1. Positive values indicate phylogenetic clustering, while negative values indicate phylogenetic overdispersion [36]. In addition, the NTI is based on the mean nearest neighbor phylogenetic distance (MNTD), which is obtained from the mean phylogenetic distance of each taxon of a given community to its nearest neighbor on a tree [46]. For both indices, positive values indicate that species co-occur more than expected by null models (phylogenetic clustering) and negative values indicate that closely related taxa co-occur less frequently than expected under a given null model (phylogenetic overdispersion). We evaluated this by 1000 iterations using ‘phylogeny.pool’; this argument randomizes the community data matrix by drawing species from the pool of species that occur in the distance matrix with equal probability [36]. We performed these analyses using the *Picante* [36] package in R ver. 4.2.3 [47], utilizing the pruned phylogeny from McGuire et al. [35].

### Statistical analyses

To evaluate whether hummingbird diversity measures were associated with floral resources, we used linear mixed models and generalized linear mixed models, in which taxonomic, functional, and phylogenetic diversity were the response variables and plant species, flower abundance, migration season, and site were the predictor variables. The number of plant species and flower abundance corresponded to the total number of plant species and number of flowers recorded in each transect of the study sites at each sampling time. We use transects to estimate temporal, taxonomic, functional, and phylogenetic diversity because this allows us to count species and individuals at 15 point counts. Migration season is when latitudinal hummingbird migratory species are recorded in the region from December to April. We used generalized linear mixed models with a gaussian distribution (identity link) for functional variables and a negative binomial distribution (log link) for taxonomic and phylogenetic variables. The sampling period and transect were included as random intercept effects, which corrects for the possibility of temporal and spatial autocorrelation, respectively. The models were calculated using the *lme4* package [48]. Normality and homogeneity of variance of the data were tested by a Shapiro–Wilk normality test, and residuals were also analyzed to assess normality [49]. We used the function Anova (package car [50]) to clarify the significance of each categorical fixed factor [49], and the Tukey test for multiple comparisons to determine the differences between fixed factors using the function glht in the package multcomp [51]. We performed all analyses in R software version 4.2.3 [47].

## Results

**Fig 1:**
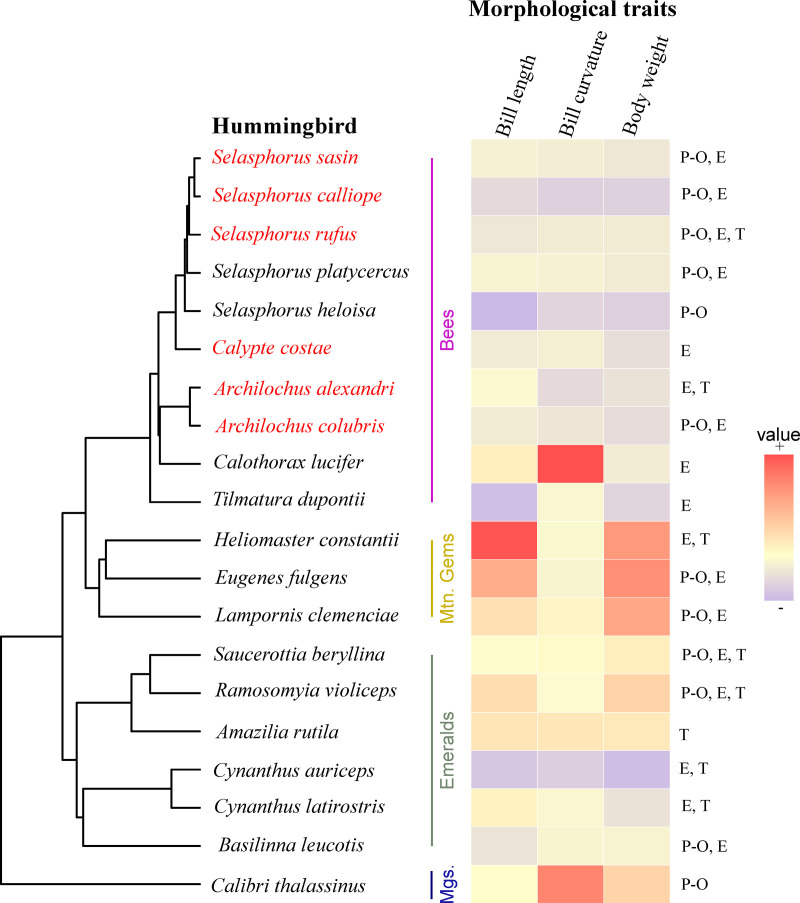
Phylogenetic tree of 20 hummingbird species and morphological traits (body weight, bill length and curvature). Latitudinal migrant species names are in red. Colored vertical labels to the right of the phylogeny indicate the four hummingbird clades (Mtn. Gems = Mountain Gems, Mgs. = Mangoes). Letters to the right of the morphological trait heat map indicate the sites where each hummingbird species was recorded (P-O: Pine-oak, E: Ecotone, and T: Tropical). Redder colors indicate higher trait values and bluer colors indicate lower ones.

We recorded 20 species of hummingbirds belonging to four clades (Fig 1) and 70 flowering plant species belonging to 30 families (S2 Table). We identified 10 hummingbird species belonging to the Bee clade, six Emeralds, three Mountain Gems, and one Mango (Fig 1). The first three clades were recorded at all sites, while the Mango clade was only recorded at the Pine-oak site (Fig 1).

**Fig 2:**
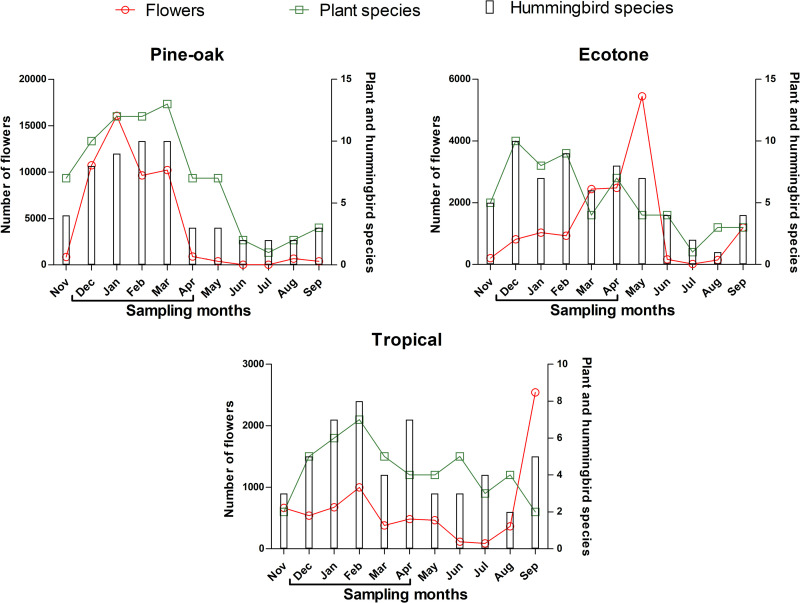
The number of flowers, plant species, and hummingbird species during the sampling months at each study site. The bars show the number of hummingbird species, the red lines the number of flowers, and the green lines the number of plant species with flowers for hummingbirds during each monthly sampling at each study site. A line below the x-axis headings denotes the months within the migratory season.

We recorded 965 individual hummingbirds along the altitudinal gradient. The pine-oak forest had the highest number of recorded hummingbirds. Hummingbird diversity changed over the course of the sampling year (Fig 2 and S1 Fig). In Pine-oak forest, hummingbird abundance followed flower abundance more than plant richness, and hummingbird richness followed both flower abundance and plant richness. In the Ecotone, hummingbird richness followed plant richness, but the peak of hummingbird richness did not coincide with the peak of flower abundance. Meanwhile, in Tropical forest, hummingbird richness followed plant richness and flower abundance throughout the year; however, peak plant and hummingbird richness did not coincide with peak flower abundance in September.

### Diversity and floral resources

Hummingbird taxonomic, functional, and phylogenetic diversity were related to the abundance of flowers hummingbirds feed on, but only phylogenetic diversity was related to plant richness (Table 1). The migratory season was positively and significantly associated with the taxonomic and phylogenetic diversity (Table 1and S2 Fig).

**Table 1:** General linear model of the taxonomic, functional, or phylogenetic α diversity of the hummingbird community as a function of migratory season, sites, plant species, and flower abundance.

	Taxonomic diversity			Functional Diversity			Phylogenetic diversity		
	*df*	*X* ^*2*^	*P*	*df*	*X* ^*2*^	*P*	*df*	*X* ^*2*^	*P*
Migratory season	1	7.791	0.005	1	2.087	0.148	1	18.942	<0.001
Sites	2	0.914	0.633	2	22.494	<0.001	2	0.535	0.765
Plant species	1	0.240	0.623	1	0.033	0.854	1	6.385	0.011
Flower abundance	1	8.871	0.002	1	6.438	0.011	1	23.619	<0.001

**Fig 3:**
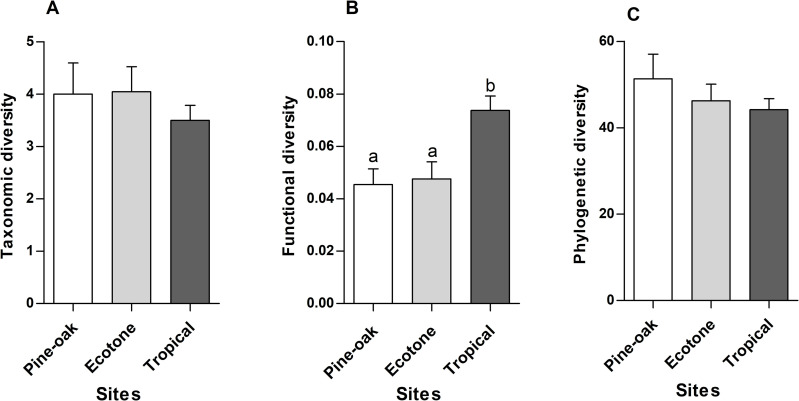
A) Taxonomic, B) functional, and C) phylogenetic of hummingbird species at each site. Tukey *post-hoc* comparison confirmed the differences among sites (P < 0.05).

Functional diversity was significantly different among sites (Table 1): Tropical forest had higher functional diversity than the Ecotone and Pine-oak forest (Tukey *post-hoc* multiple comparisons, P < 0.05; Fig 3B).

**Fig 4:**
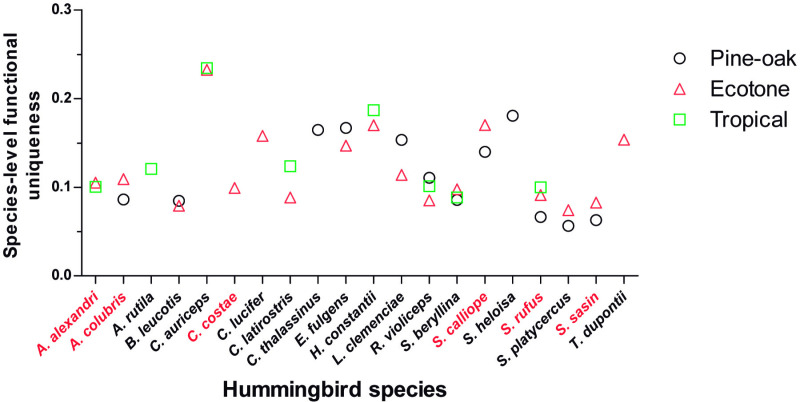
Species-level functional uniqueness for each site (Pine-oak black circles, Ecotone red triangle, and Tropical green squares). The latitudinal migratory species are indicated in red type.

*Cynanthus auriceps*, *Selasphorus heloisa*, and *Tilmatura dupontii* (the lightest and smallest-billed species), and *Heliomaster constantii*, and *Eugenes fulgens* (the heaviest and longest-billed species) had the highest species-level functional uniqueness. Meanwhile, latitudinal migratory species had low species-level functional uniqueness in the sites where they were recorded (Fig 4). The functional uniqueness of the hummingbird species was similar among sites (Fig 4). The species with the highest level of uniqueness were found at the ecotone and the tropical sites (i.e., *C. auriceps* and *H. constantii*). However, the Ecotone also had eight species with low levels of functional uniqueness, while the Pine-oak forest had six.

### Morphofunctional niche overlap

**Fig 5:**
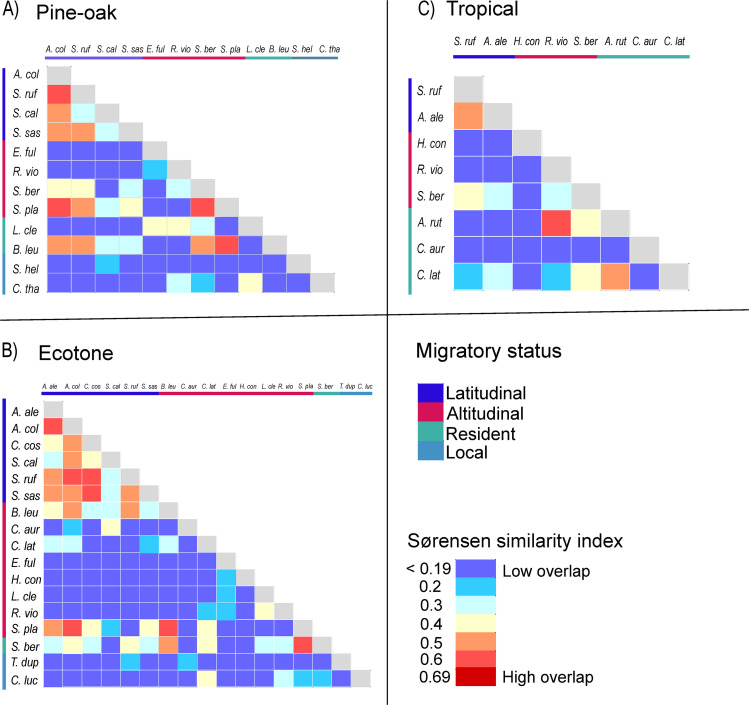
Sørensen functional similarity index between pairs of hypervolumes. **A) functional overlap of hummingbird species in Pine-oak forest; B) in Ecotone; C) in Tropical sites**.

In the PCA of functional traits, PC1 explained 59.9% of the variance, which was most related to bill length. PC2 explained 31.4% of the total variance, and bill curvature contributed the most to this axis. Finally, PC3 explained 8.6% of the total variance and the trait body weight contributed the most to this axis. The results of the hypervolume analysis showed that in the pine-oak site, there was an intermediate degree of overlap among the latitudinal migrant hummingbirds, ranging from 0.3 to 0.58. In contrast, altitudinal migrants such as *E*. *fulgens* and *R*. *violiceps* had little to no overlap with latitudinal migrants, while the resident species *S*. *beryllina* and *S*. *platycercus* had intermediate overlap with altitudinal migrants. *L*. *clemenciae*, a larger resident species, only had overlap with *E*. *fulgens* and *R*. *violiceps*. Meanwhile, *B*. *leucotis*, a medium-sized hummingbird, had overlap with several hummingbirds. Finally, the resident species *S*. *heloisa* and C. *thalassinus* had overlap <0.4 with very few species (Fig 5A). In the ecotone, which is where a greater number of species converged, an overlap of up to 0.64 was found between *S*. *rufus* and *A*. *colubris*, both latitudinal migratory species. On the other hand, *S*. *platycercus* (altitudinal migrant) and *S*. *beryllina* (resident) overlapped with most species, with values ranging from 0.05 to 0.61. Meanwhile the local species *T*. *dupontii* and *C*. *lucifer* had overlap with three and four species, < 0.42 with different species (Fig 5B). Finally, at the tropical site, the resident species *C*. *latirostris* overlapped with six species with values ranging from 0.05 to 0.50; while *H*. *constantii* (altitudinal migrant) and *C*. *auriceps* (resident) did not overlap with any species. The species *A*. *rutila* and *R*. *violiceps* showed the highest overlap with 0.62 (Fig 5C and S3 Fig).

### Phylogenetic community structure

The Pine-oak (−0.2451) and Tropical sites (−0.0525) exhibited negative NRI values, indicating that these communities are phylogenetically overdispersed. The Ecotone site hummingbird community had a positive NRI value (1.4625), showing phylogenetic clustering. The estimated NTI value was negative (−0.3429) at Pine-oak, but positive at the Ecotone (1.8774) and Tropical sites (1.2381), indicating phylogenetic clustering at those two sites.

## Discussion

Our study showed how hummingbird communities are structured around floral resources across three sites along an altitudinal gradient in northwestern Mexico. Each habitat is characterized by distinct vegetation types and climates due to differences in altitude, affecting the composition of the hummingbird communities that inhabit them. We found that the seasonality of floral resources and the latitudinal migration of hummingbirds influence taxonomic, phylogenetic, and functional diversity, as well as the morphofunctional and phylogenetic structure patterns along this gradient.

We recorded six migratory species during the latitudinal migration season (December to April), which affected taxonomic and phylogenetic diversity. The presence of latitudinal migratory hummingbirds had no effect on functional diversity, however, as migratory species exhibit similar functional morphology to resident, local, and altitudinal migrants (Fig 1). Even highly abundant latitudinal migrant species such as *S. rufus* play a redundant role within communities in Mexico, which is quickly filled by local species when they migrate [24]. On the contrary, several species that perform altitudinal/local migratory movements contribute significantly to the functional, taxonomic and phylogenetic diversity of the community. For example, some species with the highest levels of uniqueness perform altitudinal migratory movements (*H. constantii*, *E. fulgens* and *C. auriceps*); these species follow their floral resources across the different habitats along the altitudinal gradient [4,8,52]. Likewise, some species with singular traits (*S. heloisa*) or from the most phylogenetically distant clades (i.e., Mangoes, *Colibri thalassinus*) are temporary visitors to the Pine-oak site. Thus, the presence of species with unique morphological traits and different clades, such as altitudinal and local migrants, increased functional and phylogenetic variation at the study sites.

The relationship between plant richness and hummingbird functional diversity is attributed to the morphological complementarity of hummingbird bills and flowers’ corollas [28]. The importance of plant species richness is that it increases the variety of resources used by hummingbird species, which potentially favors the occurrence of hummingbird species with distinct strategies within communities [53]. However, in contrast to our expectation, we found that functional diversity was related to the flower abundance rather than to the species identity of floral resources. This pattern may reflect the generalist nature of North American hummingbirds [54], which exhibit less morphological coupling between their bills and floral structure [23]. This result is consistent with previous reports that the numerous generalist hummingbird species in temperate North America prefer abundant floral resources over a diversity of plant species [7,55].

Differences among biomes and environmental conditions is reflected in the plant communities and, therefore, hummingbird assemblages [9]. Tropical sites in the lowlands had higher functional diversity than temperate higher-altitude sites. In a study of the hummingbird community along an altitudinal gradient at EL Triunfo, Mexico, Partida-Lara et al. [56] found higher taxonomic diversity in the medium elevation site, but the functional diversity was similar among three sites at different elevations. They attributed these results to the wide distribution of some hummingbird species with redundant functional traits [56]. Our study at showed high levels of functional diversity at the Tropical site, and in consequence, low (or null) functional overlap for species such as *H*. *constantii* and *C*. *auriceps*. The morphological traits of *H. constantii* (the longest-billed species we recorded) contribute to its high functional uniqueness and reduce morphofunctional overlap in the sites where it is present. Also, this site only had two latitudinal migratory (functionally redundant) species at low abundances (*S. rufus* and *A. alexandri*).

In general, closely related taxa share more morphological traits than distantly related ones [14]. However, our results showed clustering within the Ecotone community, which was composed of species with diverse morphological traits (e.g., *H. constantii*, *C. lucifer*, and *C. auriceps*), many of which are generalists with redundant morphologies. As elevation in the tropics increases, functional and phylogenetic structures of avian assemblages tend to become uncoupled, suggesting that only some close relatives share similar trait combinations [57]. At the Ecotone site, we found phylogenetically related species with high morphofunctional overlap (Bee clade), while many species from the Mountain Gems clade exhibited low overlap. Multiple forces drive this combination of species and their phylogenetic and functional relationships in this region.

Hummingbird communities exhibit different patterns of phylogenetic composition along altitudinal gradients, with some communities displaying phylogenetic clustering (co-occurrence of closely related species) and others showing phylogenetic overdispersion (co-occurrence of distantly related species) [15]. For example, at high elevations in the Ecuadorian Andes (over 3000 m a.s.l.), hummingbird communities are phylogenetically clustered [15]. On the contrary, our study found overdispersion of the hummingbird communities at the highest-elevation site (Pine-oak). The incongruent pattern that we found could be due to the presence of few resident hummingbirds along with incoming local migrants that were not found anywhere else along the elevation gradient. For example, only two of the 12 species recorded at the Pine-oak site are residents, while the local migrant *C. thalassinus* was the only species from the Mango clade recorded in our sampling. Puga-Caballero et al. [58] proposed that biotic mixing and habitat heterogeneity across the elevation gradient in North American may explain overdispersed patterns by allowing species from different lineages to coexist. We hypothesize that in contrast to the Andes, where interconnected high-elevation ecosystems host resident hummingbird species, Mexico’s high mountain sites are discontinuous, with limited, seasonal floral resources, resulting in unique phylogenetic compositions shaped by migratory movements.

Macarthur and Levins [1] proposed that the diversity of coexisting species depends on competition between species with similar morphologies and niche specialization. In hummingbird communities, similarity in morphological traits can increase competition for the same floral resources due to overlapping functional niches [59]. Our results showed that high (Pine-oak) and mid-elevation (Ecotone) sites have many species that overlap in morphological and functional space, and compete for the same type of floral resources in the region [23,26]. López-Segoviano et al. [23,26] found that competition for floral resources and hummingbird morphology structure the feeding niches of hummingbirds and their flowers in Pine-oak site. Competition for the availability and amount of food resources organizes hummingbird communities whose species share similar feeding niches [12,60]. The most dominant species displace the less dominant species to flower patches or plants with low available energy [12,60]. Thus, the abundance of floral resources and feeding behavior may play important roles in North America’s assembly of hummingbirds and their floral resources [19,23,26]. In contrast, in the tropical lowlands of northern South America, the lack of overlap in morphofunctional space among co-occurring hummingbird species may not result from competition, but rather from independent evolutionary trajectories, in which behaviors and morphologies are already differentiated, allowing them to coexist [14].

Recent studies have found that climate change and land-use change will seriously impact individual species and communities of hummingbirds throughout Mexico in the coming decades [61]. Although montane regions may face less direct impacts, biotic composition is expected to change as species shift their elevational ranges, potentially resulting in range contractions for some species [61]. Furthermore, changes in the phenology of hummingbird-pollinated plants may alter community structure, functional diversity, and reproductive success in the future [8,62]. Plants pollinated by hummingbirds are also likely to undergo changes in geographical distribution and flowering phenology under different climate scenarios [62], and such variation may impact the community structure, functional diversity, ecological networks, and reproductive success of plants in the future. Lastly, long-distance migratory species, such as *Selasphorus rufus*, *S. calliope*, and *Archilochus colubris*, migrate in synchrony with blooming patterns along their routes and may be unable to adjust their migration timing if these patterns shift [63]. Therefore, the fluctuations in the availability of floral resources are more critical to the long-distance migratory species than other hummingbird species in the region.

## Conclusion

Hummingbirds respond to changes in food resources by performing local and regional migratory movements, which directly influence species composition and functions. Latitudinal hummingbird migration does not increase the overall functional diversity of our study system because migratory species perform redundant roles. Seasonal mountain flowering influences hummingbird migration, species’ functional roles and occurrence of diverse phylogenetic clades. Our study shows that the structure of hummingbird communities in the mountains of northwestern Mexico is driven by seasonal floral resources and complex migratory behavior of hummingbird species.

## Supporting information

S1 Table Migratory status (resident, latitudinal, altitudinal and local migratory), and abundance of the hummingbird species at each study site. (DOCX)

S2 Table Order, family, and flowers abundance of the plant species visited by hummingbirds at each study site. (DOCX)

S1 Fig The number of flowers, plant species, and number of hummingbirds during the sampling months at each study site. The bars show the number of hummingbirds, the red lines the number of flowers, and the green lines the number of plant species with flowers for hummingbirds during each month’s samplings at each study site. A line below the x-axis headings denotes the months within the migratory season.
(DOCX)

S2 Fig A) Taxonomic, B) functional, and C) phylogenetic of hummingbird species at each season. Tukey *post-hoc* multiple comparisons a posteriori comparison test confirmed the differences among Sites (P < 0.05).
(DOCX)

S3 Fig Morphological space by site. **A) Pine-oak; B) in Ecotone; C) Tropical. Each hypervolume corresponds to one species and the colors indicate the migratory status**.
(DOCX)
