# Supplementary material for: Flowering seasonality drives taxonomic, functional, and phylogenetic diversity of hummingbirds along an altitudinal gradient in northwestern Mexico
Source: PLoS One. 2025 Jun 11;20(6):e0324881. doi: 10.1371/journal.pone.0324881 (PMC12156984; doi:10.1371/journal.pone.0324881)
Supplement: S1 Table — (DOCX) [file pone.0324881.s001.docx]

S1 Table. Migratory status (resident, latitudinal, altitudinal and local migratory), and abundance of the hummingbird species at each study site.

| Hummingbird Specie | Site | Migratory status | Total of hummingbirds |
| --- | --- | --- | --- |
| *Archilochus colubris* | Pine-oak | Latitudinal | 1 |
| *Basilinna leucotis* | Pine-oak | Resident | 212 |
| *Colibri thalassinus* | Pine-oak | Local | 12 |
| *Eugenes fulgens* | Pine-oak | Altitudinal | 14 |
| *Lampornis clemenciae* | Pine-oak | Resident | 44 |
| *Ramosomyia violiceps* | Pine-oak | Altitudinal | 1 |
| *Saucerottia beryllina* | Pine-oak | Altitudinal | 31 |
| *Selasphorus calliope* | Pine-oak | Latitudinal | 2 |
| *Selasphorus heloisa* | Pine-oak | Local | 6 |
| *Selasphorus platycercus* | Pine-oak | Altitudinal | 11 |
| *Selasphorus. rufus* | Pine-oak | Latitudinal | 84 |
| *Selasphorus sasin* | Pine-oak | Latitudinal | 2 |
| *Archilochus alexandri* | Ecotone | Latitudinal | 1 |
| *Archilochus colubris* | Ecotone | Latitudinal | 3 |
| *Basilinna leucotis* | Ecotone | Altitudinal | 16 |
| *Calypte costae* | Ecotone | Latitudinal | 2 |
| *Cynanthus latirostris* | Ecotone | Altitudinal | 10 |
| *Calothorax lucifer* | Ecotone | Local | 2 |
| *Eugenes fulgens* | Ecotone | Altitudinal | 1 |
| *Heliomaster constantii* | Ecotone | Altitudinal | 2 |
| *Lampornis clemenciae* | Ecotone | Altitudinal | 12 |
| *Ramosomyia violiceps* | Ecotone | Altitudinal | 12 |
| *Saucerottia beryllina* | Ecotone | Resident | 156 |
| *Selasphorus calliope* | Ecotone | Latitudinal | 9 |
| *Selasphorus platycercus* | Ecotone | Altitudinal | 8 |
| *Selasphorus rufus* | Ecotone | Latitudinal | 15 |
| *Selasphorus sasin* | Ecotone | Latitudinal | 1 |
| *Tilmatura dupontii* | Ecotone | Local | 1 |
| *Archilochus alexandri* | Tropical | Resident | 6 |
| *Amazilia rutila* | Tropical | Latitudinal | 80 |
| *Cynanthus auriceps* | Tropical | Resident | 15 |
| *Cynanthus latirostris* | Tropical | Resident | 154 |
| *Heliomaster constantii* | Tropical | Altitudinal | 20 |
| *Ramosomyia violiceps* | Tropical | Altitudinal | 9 |
| *Saucerottia beryllina* | Tropical | Altitudinal | 3 |
| *Selasphorus rufus* | Tropical | Latitudinal | 6 |
