# Supplementary material for: Flowering seasonality drives taxonomic, functional, and phylogenetic diversity of hummingbirds along an altitudinal gradient in northwestern Mexico
Source: PLoS One. 2025 Jun 11;20(6):e0324881. doi: 10.1371/journal.pone.0324881 (PMC12156984; doi:10.1371/journal.pone.0324881)
Supplement: S2 Table — (DOCX) [file pone.0324881.s002.docx]

S2 Table. Order, family, and flowers abundance of the plant species visited by hummingbirds at each study site.

|  |  |  | Number of flowers | | |
| --- | --- | --- | --- | --- | --- |
| Orden | Family | Specie | Pine-oak | Ecotone | Tropical |
| *Lamiales* | Bignoniaceae | *Adenocalymma inundatum* |  |  | 30 |
| *Asparagales* | Asparagaceae | *Agave inaequidens subsp. Barrancensis* | 1860 |  |  |
| *Asparagales* | Asparagaceae | *Agave maximiliana* | 1745 |  |  |
| *Caryophyllales* | Polygonaceae | *Antigonon leptopus* |  |  | 3214 |
| *Ericales* | Ericaceae | *Arbutus sp.* | 325 |  |  |
| *Lamiales* | Bignoniaceae | *Bignonia aequinoctialis* |  |  | 4 |
| *Gentianales* | Rubiaceae | *Bouvardia tenuifolia* | 604 |  |  |
| *Poales* | Bromeliaceae | *Bromelia pinguin* |  |  | 14 |
| *Fabales* | Fabaceae | *Calliandra houstoniana var. Anomala* |  | 22 |  |
| *Fabales* | Fabaceae | *Calliandra laevis* |  | 12 |  |
| *Fabales* | Fabaceae | *Canavalia villosa* |  | 753 |  |
| *Zingiberales* | Cannaceae | *Canna indica* |  | 147 |  |
| *Lamiales* | Orobanchaceae | *Castilleja arvensis* | 222 | 8 |  |
| *Lamiales* | Orobanchaceae | *Castilleja tenuiflora* | 468 | 21 |  |
| *Malvales* | Malvaceae | *Ceiba aesculifolia subsp. aesculifolia* |  |  | 32 |
| *Solanales* | Solanaceae | *Cestrum thyrsoideum* | 7462 |  |  |
| *Myrtales* | Combretaceae | *Combretum fruticosum* |  |  | 76 |
| *Lamiales* | Boraginaceae | *Cordia alliodora* |  |  | 214 |
| *Lamiales* | Bignoniaceae | Crescentia alata |  |  | 114 |
| *Myrtales* | Lythraceae | *Cuphea hookeriana* | 153 | 221 |  |
| *Myrtales* | Lythraceae | *Cuphea sp.* |  | 613 |  |
| *Myrtales* | Lythraceae | *Cuphea watsoniana* | 747 |  |  |
| *Fabales* | Fabaceae | *Erythrina flabelliformis* |  | 221 |  |
| *Fabales* | Fabaceae | *Erythrina lanata* |  |  | 506 |
| *Gentianales* | Rubiaceae | *Fuchsia cylindracea* | 376 |  |  |
| *Gentianales* | Apocynaceae | *Gonolobus sp.* |  |  | 214 |
| *Gentianales* | Rubiaceae | *Hamelia versicolor* |  |  | 227 |
| *Lamiales* | Bignoniaceae | *Handroanthus impetiginosus* |  |  | 210 |
| *Fabales* | Fabaceae | *Inga vera* |  | 6144 |  |
| *Solanales* | Convolvulaceae | *Ipomoea arborescens* |  |  | 551 |
| *Solanales* | Convolvulaceae | *Ipomoea bracteata* |  |  | 1389 |
| *Solanales* | Convolvulaceae | *Ipomoea dumosa* |  | 392 |  |
| *Solanales* | Convolvulaceae | *Ipomoea hederifolia* |  | 108 |  |
| *Solanales* | Convolvulaceae | *Ipomoea murucoides* |  | 340 |  |
| *Lamiales* | Acanthaceae | *Justicia candicans* |  |  | 74 |
| *Lamiales* | Acanthaceae | *Justicia salviiflora* |  | 21 |  |
| *Asterales* | Asteraceae | Lagascea helianthifolia | 72 |  |  |
| *Lamiales* | Scrophulariaceae | Lamourouxia rhinanthifolia | 89 |  |  |
| *Lamiales* | Verbenaceae | *Lantana camara* |  | 108 |  |
| *Lamiales* | Lamiaceae | *Leonotis nepetifolia* |  | 44 |  |
| *Asterales* | Campanulaceae | *Lobelia laxiflora* | 323 | 70 |  |
| *Ericales* | Polemoniaceae | *Loeselia mexicana* | 577 |  |  |
| *Myrtales* | Onagraceae | *Lopezia semeiandra* |  | 4110 |  |
| *Lamiales* | Gesneriaceae | *Moussonia jaliscana* |  | 55 |  |
| *Solanales* | Convolvulaceae | *Operculina pteripes* |  |  | 200 |
| *Caryophyllales* | Cactaceae | *Pachycereus pecten-aboriginum* |  |  | 2 |
| *Santalales* | Santalaceae | *Phoradendron sp.* | 10 |  |  |
| *Cucurbitales* | Cucurbitaceae | *Polyclathra cucumerina* |  |  | 100 |
| *Malvales* | Malvaceae | *Pseudobombax palmeri* |  | 7 | 105 |
| *Santalales* | Loranthaceae | *Psittacanthus macrantherus* | 2168 |  |  |
| *Santalales* | Loranthaceae | *Psittacanthus ramiflorus* |  | 695 |  |
| *Lamiales* | Plantaginaceae | *Russelia coccinea* |  |  | 274 |
| *Lamiales* | Plantaginaceae | *Russelia elongata* |  | 734 |  |
| *Lamiales* | Plantaginaceae | *Russelia furfuracea* |  |  | 44 |
| *Lamiales* | Plantaginaceae | *Russelia tetraptera* |  | 110 |  |
| *Lamiales* | Lamiaceae | *Salvia elegans* | 4526 |  |  |
| *Lamiales* | Lamiaceae | *Salvia gesneriiflora* | 669 |  |  |
| *Lamiales* | Lamiaceae | *Salvia iodantha* | 27051 |  |  |
| *Lamiales* | Lamiaceae | *Salvia lavanduloides* | 100 |  |  |
| *Lamiales* | Lamiaceae | *Salvia mexicana* | 535 | 25 |  |
| *Lamiales* | Lamiaceae | *Salvia roscida* |  | 206 |  |
| *Lamiales* | Lamiaceae | *Stachis sp.* |  | 57 |  |
| *Lamiales* | Lamiaceae | *Stachys coccinea* | 56 |  |  |
| *Gentianales* | Apocynaceae | *Stemmadenia tomentosa* |  |  | 49 |
| *Caryophyllales* | Cactaceae | *Stenocereus alamosensis* |  |  | 61 |
| *Poales* | Bromeliaceae | *Tillandsia bourgaei* | 129 |  |  |
| *Poales* | Bromeliaceae | *Tillandsia caput-medusae* |  |  | 48 |
| *Poales* | Bromeliaceae | *Tillandsia fasciculata* |  |  | 27 |
| *Poales* | Bromeliaceae | *Tillandsia recurvata* |  |  | 3 |
| *Malvales* | Malvaceae | *Triumfetta sp.* | 8 | 95 |  |
