# Supplementary figures and images for: Flowering seasonality drives taxonomic, functional, and phylogenetic diversity of hummingbirds along an altitudinal gradient in northwestern Mexico
Source: PLoS One. 2025 Jun 11;20(6):e0324881. doi: 10.1371/journal.pone.0324881 (PMC12156984; doi:10.1371/journal.pone.0324881)

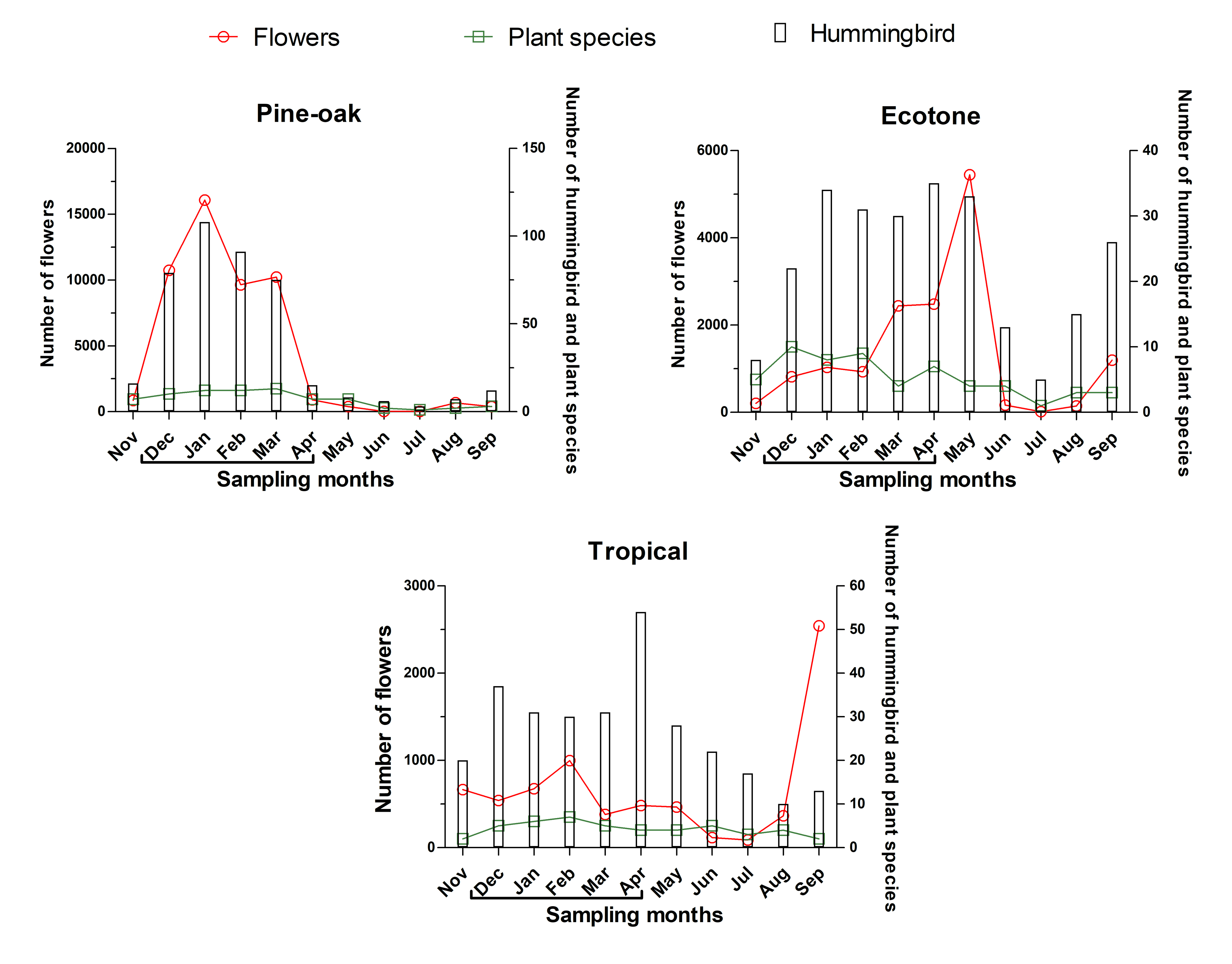

Supplement: S1 Fig — The bars show the number of hummingbirds, the red lines the number of flowers, and the green lines the number of plant species with flowers for hummingbirds during each month’s samplings at each study site. A line below the x-axis headings denotes the months within the migratory season. (DOCX) [file pone.0324881.s003.tif]

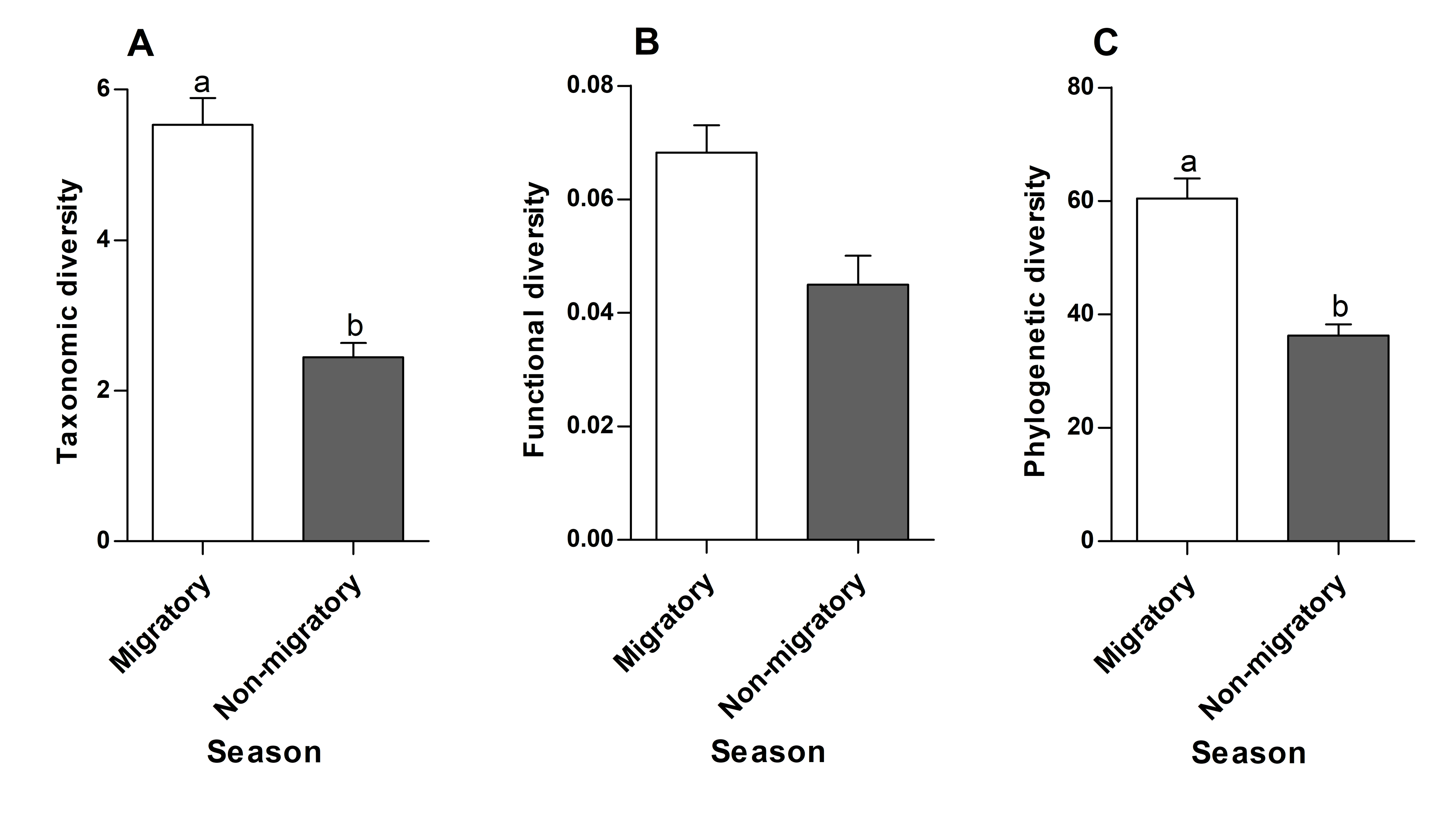

Supplement: S2 Fig — Tukey post-hoc multiple comparisons a posteriori comparison test confirmed the differences among Sites (P < 0.05). (DOCX) [file pone.0324881.s004.tif]

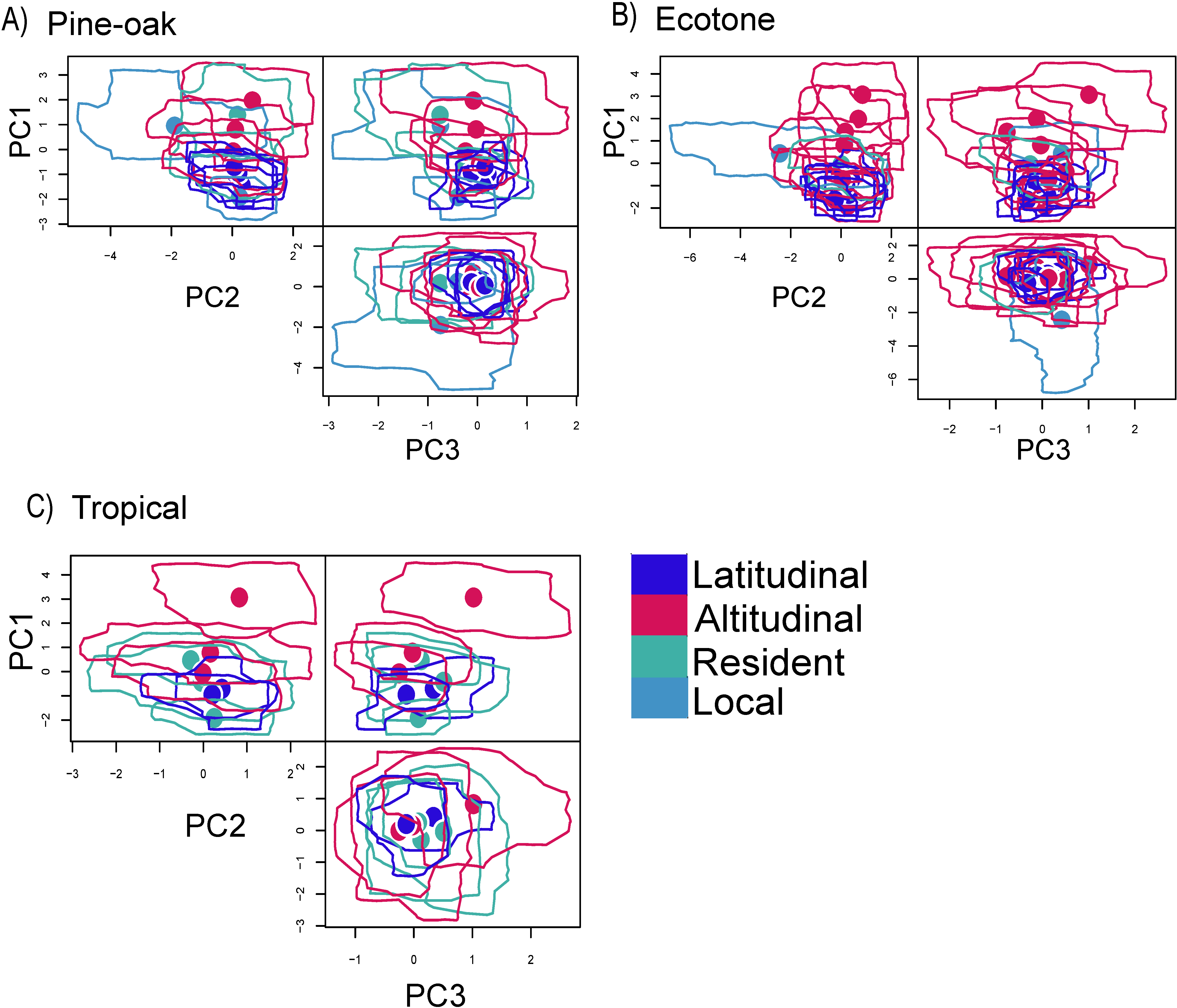

Supplement: S3 Fig — A) Pine-oak; B) in Ecotone; C) Tropical. Each hypervolume corresponds to one species and the colors indicate the migratory status. (DOCX) [file pone.0324881.s005.tif]
